# Recognizing a Non-classical Telomeropathy before Hematopoietic Stem Cell Transplantation in Pediatric Patients: A Case Series

**DOI:** 10.1097/HS9.0000000000000282

**Published:** 2019-07-05

**Authors:** Iris Nederlof, Caroline A. Lindemans, Marc B. Bierings, Alexander B. Mohseny, Dorine Bresters, Marije Bartels

**Affiliations:** 1 Department of Pediatric Hematology, University Medical Center (UMC) Utrecht, Utrecht, The Netherlands.; 2 Princess Máxima Center for Pediatric Oncology, Utrecht, The Netherlands; 3 Department of Stem Cell Transplantation, Wilhelmina Children's Hospital, University Medical Center (UMC) Utrecht, Utrecht, the Netherlands; 4 Willem-Alexander Children's Hospital, Department of Stem Cell Transplantation, Leiden University Medical Center, Leiden, The Netherlands.

The nucleoprotein structures located at the ends of chromosomes are telomeres and protect genetic information from damage and attrition. Aberrations of telomere maintenance and the cellular response to unprotected and shortened telomeres cause telomeropathy and comprise a diverse spectrum of diseases.^1^ Telomeropathies are characterized by bone marrow failure (BMF), lung fibrosis, liver fibrosis (and cirrhosis), and cancer predisposition.^2–4^ Short telomere length was demonstrated in children with dyskeratosis congenita (DC) and in a subset of individuals with severe aplastic anemia (SAA) already 20 years ago.^5–7^ DC is associated with extremely short telomere length (<p1), and age-adjusted values for telomere length provide a quantitative measure of disease severity.^8^ Hematopoietic stem cell transplantation (HSCT) for telomeropathies is associated with an increased risk of transplant-related complications, predominantly progressive organ dysfunction, and tissue damage (eg, mucositis) following conditioning regimen.^9,10^ Therefore, treating patients with a telomeropathy requires the adjustment of HSCT protocols, including reduced intensity conditioning (RIC) regimen and screening of potentially affected (and asymptomatic) family donors.^11–13^ Screening for telomeropathies is of particular importance in young patients, as the disorder encompasses a wide spectrum of clinical problems and risks for therapy, while the phenotype may be subtle. Currently, telomere length analysis in patients suffering from bone marrow failure is not standard procedure, which can lead to delayed or missed diagnosis of a telomeropathy. Here, we describe 6 pediatric cases illustrating the diagnostic pitfalls and clinical challenges in patients suffering from a non-classical telomeropathy with respect to the outcome of allogeneic HSCT (summarized in Table 1**)**.

**Table 1:**
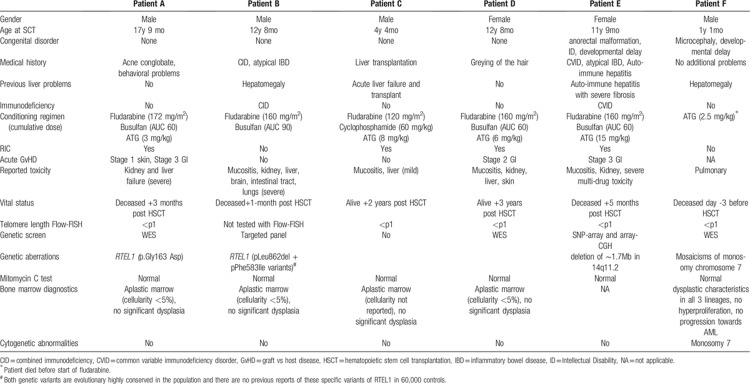
Clinical Characteristics of Patients A–F.

**Patient A,** a 17-year-old male, was diagnosed with SAA. His medical history was characterized by acne conglobata, behavioral problems, and disturbed liver enzymes in the previous year. Telomere length analysis demonstrated very short telomeres (Table 1). Organ screening showed no signs of liver or lung fibrosis specifically. He was scheduled for HSCT from a matched unrelated donor (MUD) following reduced intensity conditioning (RIC) (Table 1). Engraftment was established at day 21 (95% donor signal). The first month after HSCT was characterized by transient kidney failure and hyperbilirubinemia; abdominal ultrasound analysis suggested liver cirrhosis, followed by liver biopsy showing moderate to severe fibrosis and no signs of graft versus host disease (GvHD). Subsequently, he developed GvHD grade III with involvement of the intestinal tract (skin stage 1). The combination of treatment-refractory GvHD and progressive liver failure led to death of the patient 3 months after HSCT. Post mortem genetic analysis on pre-transplantation blood samples revealed a pathogenic DC mutation in RTEL.

**Patient B** was diagnosed with combined immunodeficiency (CID), atypical inflammatory bowel disease (IBD), and failure to thrive. At the age of 12 years, he developed severe bone marrow failure (cellularity <5%). Pre-HSCT screening tests demonstrated inhomogeneous hepatomegaly and elevated liver enzymes, and minor bronchopathy on the HR-CT, explained by chronic infections. There were no signs of lung fibrosis. He was scheduled for HSCT from an HLA-identical sibling following a myeloablative conditioning regimen (Table 1) and neutrophil engraftment was established at day 24 (100% donor signal). In the following weeks, he developed severe multi-organ toxicity, including mucositis and transient kidney failure. Subsequently, a rapid deterioration of liver tests (bilirubin >500 μmol/L) prompted liver biopsy, demonstrating non-specific (toxic) damage. Next, he showed neurological deterioration, accompanied by white matter abnormalities on MRI. One month after HSCT, he died of progressive multi-organ failure. Post-mortem examination revealed extensive organ damage to the liver, intestinal tract, brains and lungs. Genetic analysis revealed compound heterozygous mutations in *RTEL1*.

**Patient C**, a 4-year-old boy, developed acute liver failure of unknown cause requiring a liver transplant. Three months after liver transplantation he was diagnosed with severe acquired aplastic anemia and was scheduled for MUD-HSCT. Screening pre-SCT revealed normal liver function, and no signs of lung fibrosis. Telomere length analysis revealed very short telomeres, suggesting an underlying telomeropathy. Additional genetic analysis was not performed. HSCT was performed following RIC conditioning (Table 1). Engraftment was established at day 16 with 100% donor signal. Early post-SCT follow up was uneventful. In the second year after SCT, he was treated with a second liver transplantation after liver graft rejection. Since then he is in good clinical condition.

**Patient D,** a 12-year-old girl, was diagnosed with SAA. A pre-SCT work-up revealed no abnormalities of lungs and liver. Subtle greying of a part of her hair in combination with SAA prompted telomere length analysis, revealing very short telomeres. Genetic screening for DC was negative. She was scheduled for MUD-HSCT following a RIC regimen (Table 1). Engraftment was established at day 15 (97% donor signal). After HSCT, she developed severe mucositis and transient kidney failure followed by acute GvHD of the intestinal tract and liver (overall grade III), treated with prednisone. In addition, she developed skin lesions (scaling, hyperpigmentation), which were attributed to her underlying telomeropathy. Persistent abnormal liver tests normalized without intervention, and topical steroids supported recovery of the skin. Three years after SCT, she is in good clinical condition.

**Patient E,** an 11-year old girl, suffered from combined variable immunodeficiency (CVID) accompanied by auto-immune phenomena, including cytopenias, atypical IBD and hepatitis (with severe fibrosis). Her medical history furthermore revealed premature birth, anorectal malformation, psychomotor retardation and epilepsy in the context of a 14q11.2 deletion syndrome (Table 1). Based on the clinical symptomatology, a functional telomere defect was considered, which was confirmed by the presence of very short telomeres. Because of progressive immune dysregulation and liver failure, she was scheduled for HSCT from a cord blood donor, following RIC conditioning (Table 1). Engraftment was established at day 14 with 89% donor chimerism. The first month post-SCT, a mild deterioration of kidney and liver function tests was observed. After 4 months, she developed severe GvHD of the intestinal tract in combination with multi-drug toxicity, eventually resulting in severe bleeding complications (intestinal), sepsis and acute respiratory distress syndrome (ARDS), leading to fatal multi-organ failure. She died five months after SCT.

**Patient F**, a 13-month old boy, was previously diagnosed with microcephaly (no genetic disorder identified with WES), developmental delay, and mosaicism of chromosome 7 monosomy. At the age of 3 months, macrocytic anaemia and mild thrombocytopenia were diagnosed, which developed into pancytopenia the following months, classified as refractory cytopenia of childhood (RCC), and was accompanied by unexplained hepatomegaly. Based on the combination of clinical features, an underlying telomeropathy was suspected, which was confirmed by telomere length analysis (Table 1). He was scheduled for HSCT from a cord blood donor, but during day 2 of the ATG, he developed a fever and respiratory insufficiency. Within 3 days his clinical condition deteriorated and he developed irreversible circulatory failure caused by a pulmonary hypertensive crisis, leading to the death of the patient.

In this report, we describe 6 pediatric patients treated with HSCT in the context of an underlying telomeropathy other than the classical DC phenotype. We illustrate that telomeropathies can be characterized by a variety of clinical characteristics, including BMF, congenital anomalies, liver failure and fibrosis, and immunodeficiency. Two out of 6 patients had a *RTEL1* mutation, both diagnosed post-mortem, and potentially associated with a severe disease phenotype. Pre-existent liver dysfunction (elevated liver enzymes, abnormal radiological aspect) was reported for 4 out of 6 telomeropathy patients (Table 1), and severe congenital abnormalities were present in 2 patients. As telomeropathy is associated with pulmonary fibrosis, abnormalities on the HR-CT of the lungs associated with fibrosis were assessed in all patients but were not found. The high overall toxicity and treatment related mortality in patients indicates that screening for telomere disease is important in order to optimize HSCT procedures and conditioning regimen. The most severe toxicity was observed in patient B, who received a myeloablative conditioning regimen. The 2 telomeropathy patients with a good clinical outcome after transplant (C and D) both received a RIC regimen, indicating that a timely diagnosis of telomeropathy is crucial to adjust treatment regimens. Concurrent immunodeficiency and severe immune-mediated colitis and hepatitis were observed in patients B and E. Immunodeficiency followed by severe BMF has been reported in previously described telomere diseases, including Hoyeraal–Hreidarsson syndrome and Revesz syndrome.^11^ Interestingly, a recent study showed that mutations in the telomerase complex genes directly affect the development and function of the T-lymphocyte compartment, inducing primary T-cell immunodeficiency in telomeropathies.^14^ Importantly, it has been recently demonstrated that that telomere length analysis in patient care has a good diagnostic specificity for young patients and can guide genetic counseling and clinical symptom assessment.^15^ From our data, we would recommend that for patients with unexplained BMF and/or immunodeficiency, pre-HSCT screening tests should include telomere length analysis regardless of classic DC symptoms. Symptoms of telomere disease can be subtle, yet transplant related morbidity in these patients may be severe. Further studies to define the complete telomeropathy spectrum in combination with efforts to uncover the underlying cellular processes driving clinical symptomatology are needed to improve diagnostics and overall clinical care for this patient group.

## References

[1] Blackburn EH Epel ES Lin J Human telomere biology: A contributory and interactive factor in aging, disease risks, and protection. *Science.* 2015;350:1193–1198. 26785477 10.1126/science.aab3389

[2] Armanios MY Chen JJ-L Cogan JD Telomerase mutations in families with idiopathic pulmonary fibrosis. *N Engl J Med.* 2007;356:1317–1326. 17392301 10.1056/NEJMoa066157

[3] Calado RT Regal JA Kleiner DE A spectrum of severe familial liver disorders associate with telomerase mutations. *PLoS One.* 2009;4:e7926. 19936245 10.1371/journal.pone.0007926 PMC2775683

[4] Townsley DM Dumitriu B Young NS Bone marrow failure and the telomeropathies. *Blood.* 2014;124:2775–2783. 25237198 10.1182/blood-2014-05-526285 PMC4215309

[5] Mitchell JR Wood E Collins K A telomerase component is defective in the human disease dyskeratosis congenita. *Nature.* 1999;402:551–555. 10591218 10.1038/990141

[6] Ball SE Gibson FM Rizzo S Progressive telomere shortening in aplastic anemia. *Blood.* 1998;91:3582–3592. 9572992

[7] Brümmendorf TH Maciejewski JP Mak J Telomere length in leukocyte subpopulations of patients with aplastic anemia. *Blood.* 2001;97:895–900. 11159514 10.1182/blood.v97.4.895

[8] Alter BP Rosenberg PS Giri N Telomere length is associated with disease severity and declines with age in dyskeratosis congenita. *Haematologica.* 2012;97:353–359. 22058220 10.3324/haematol.2011.055269 PMC3291588

[9] Bacigalupo A How I treat acquired aplastic anemia. *Blood.* 2017;129:1428–1436. 28096088 10.1182/blood-2016-08-693481

[10] Dietz AC Orchard PJ Baker KS Disease-specific hematopoietic cell transplantation: nonmyeloablative conditioning regimen for dyskeratosis congenita. *Bone Marrow Transpl.* 2011;46:98–104. 10.1038/bmt.2010.65 PMC9341256 20383216

[11] Armanios M Blackburn EH The telomere syndromes. *Nat Rev Genet.* 2012;13:693–704. 22965356 10.1038/nrg3246 PMC3548426

[12] de la Fuente J Dokal I Dyskeratosis congenita: advances in the understanding of the telomerase defect and the role of stem cell transplantation. *Pediatr Transpl.* 2007;11:584–594. 10.1111/j.1399-3046.2007.00721.x 17663679

[13] Savage SA Dokal I Armanios M Dyskeratosis congenita: the first NIH clinical research workshop. *Pediatr Blood Cancer.* 2009;53:520–523. 19415736 10.1002/pbc.22061 PMC2739803

[14] Jose SS Tidu F Burilova P The telomerase complex directly controls hematopoietic stem cell differentiation and senescence in an induced pluripotent stem cell model of telomeropathy. *Front Genet.* 2018;9:345. 30210531 10.3389/fgene.2018.00345 PMC6123533

[15] Alder JK Hanumanthu VS Strong MA Diagnostic utility of telomere length testing in a hospital-based setting. *Proc Natl Acad Sci U S A.* 2018;115:E2358–E2365. 29463756 10.1073/pnas.1720427115 PMC5877993

